# Complete Genome Sequence of a Novel Recombinant GII.Pe_GII.17 Norovirus Strain from Hong Kong in 2015

**DOI:** 10.1128/genomeA.01338-15

**Published:** 2015-11-12

**Authors:** Martin C. W. Chan, Tin-Nok Hung, Kirsty Kwok, Paul K. S. Chan

**Affiliations:** Department of Microbiology, Faculty of Medicine, Chinese University of Hong Kong, Hong Kong Special Administrative Region, People’s Republic of China

## Abstract

The complete genome sequence of a novel recombinant GII.Pe_GII.17 norovirus strain, tentatively named GII.17 Hong Kong 2015, was determined. RNA-dependent RNA polymerase has 95.6% and 98.4% and viral protein 1 has 90.6% and 95.9% identity at the nucleotide and amino acid levels, respectively, to the closest sequences in GenBank.

## GENOME ANNOUNCEMENT

Norovirus is a leading cause of acute gastroenteritis in all age groups worldwide (1). Genetically, norovirus is classified into at least 6 genogroups which are further subdivided into over 40 genotypes (2). In the winter of 2014–2015, a novel variant of norovirus genogroup II genotype 17 (GII.17), called Kawasaki 2014, emerged and became predominant in Asia (3–6) and was sporadically detected in the United States (7) and Europe (8). Genetic recombination has been proposed as one mechanism behind the emergence of novel norovirus variants (9). Here, we report the complete genome sequence of a novel recombinant norovirus strain with the RNA-dependent RNA polymerase (RdRp) closely related to GII.Pe of the current pandemic variant GII.4 Sydney 2012 and the viral protein 1 (VP1) belonging to the GII.17 genotype but distantly related to all known GII.17 variants. The norovirus GII.17 genotype is undergoing rapid evolution and intergenotypic recombination.

This recombinant norovirus strain (GII/Hu/HKG/2015/GII.Pe_GII.17/CUHK-NS-682) was retrieved from a stool specimen of a 63-year-old man on 30 June 2015 in our ongoing, hospital-based norovirus surveillance in Hong Kong (10, 11). Viral RNA was extracted from 10% stool suspension using a MagMAX viral RNA isolation kit (Thermo Scientific). Conversion of viral RNA to cDNA was performed using SuperScript III reverse transcriptase (Thermo Scientific) with a tagged oligo dT primer. Seven overlapping PCR fragments covering the whole genome were amplified using high fidelity Phusion DNA polymerase (Thermo Scientific), followed by Sanger sequencing. DNA chromatograms were manually curated, and contigs were generated using ChromasPro software version 1.7.6 for Windows (Technelysium). Primers used were available upon request.

Norovirus genotyping was performed using NoroNet’s norovirus genotyping tool (http://www.rivm.nl/mpf/norovirus/typingtool). Preliminary analysis of our norovirus strain indicated that the genotypes of RdRp and VP1 belonged to GII.Pe and GII.17, respectively, suggesting an intergenotypic recombinant. BLAST analysis was performed on RdRp and VP1 separately. The recombinant’s RdRp GII.Pe had the highest nucleotide (95.6%) and amino acid (98.4%) identity to Hu/GII/BG1C0391/2012/BGD (GenBank accession no. KJ685406), which was a GII.4 Sydney 2012 strain. Interestingly, the recombinant’s GII.17 VP1 was distantly related to the recently emerged Kawasaki 2014 variant (Hu/GII/JP/2015/GII.P17_GII.17/Kawasaki308; GenBank accession no. LC037415) with only 77.0% and 86.7% identity at the nucleotide and amino acid levels, respectively. Instead, the recombinant’s GII.17 VP1 had the highest nucleotide (90.6%) and amino acid (95.9%) identity to a GII.17 strain dating back to nearly 40 years ago (Hu/GII.17/C142/GF/1978; GenBank accession no. JN699043). Notably, the observed VP1 amino acid difference of ~4% with the closest GII.17 VP1 warrants the assignment of our recombinant norovirus strain as a new GII.17 variant, tentatively named GII.17 Hong Kong 2015. Overall, the complete genome of the recombinant norovirus shared the highest nucleotide identity of 90.3% to Hu/GII.17/C142/GF/1978.

This is the first complete genome sequence of a novel recombinant GII.Pe_GII.17 norovirus. Our data indicate that norovirus GII.17 is undergoing rapid evolution and intergenotypic recombination, providing important insights into GII.17 evolution. Acquisition of the RdRp gene from the current pandemic GII.4 Sydney 2012 variant highlights the epidemic or even pandemic potential of norovirus GII.17.

### Nucleotide sequence accession number.

The complete genome sequence of the recombinant norovirus strain GII/Hu/HKG/2015/GII.Pe_GII.17/CUHK-NS-682 has been deposited in GenBank under the accession number KT589391.

## References

[1] Ahmed SM, Hall AJ, Robinson AE, Verhoef L, Premkumar P, Parashar UD, Koopmans M, Lopman BA 2014 Global prevalence of norovirus in cases of gastroenteritis: a systematic review and meta-analysis. Lancet Infect Dis 14:725–730. doi:10.1016/S1473-3099(14)70767-4. 24981041 PMC8006533

[2] Vinjé J 2015 Advances in laboratory methods for detection and typing of norovirus. J Clin Microbiol 53:373–381. doi:10.1128/JCM.01535-14. 24989606 PMC4298492

[3] Matsushima Y, Ishikawa M, Shimizu T, Komane A, Kasuo S, Shinohara M, Nagasawa K, Kimura H, Ryo A, Okabe N, Haga K, Doan Y, Katayama K, Shimizu H 2015 Genetic analyses of GII.17 norovirus strains in diarrheal disease outbreaks from December 2014 to Mart 2015 in Japan reveal a novel polymerase sequence and amino acid substitutions in the capsid region. Eurosurveillance 20:21173. doi:10.2807/1560-7917.ES2015.20.26.21173. 26159307

[4] Lu J, Sun L, Fang L, Yang F, Mo Y, Lao J, Zheng H, Tan X, Lin H, Rutherford S, Guo L, Ke C, Hui L 2015 Gastroenteritis outbreaks caused by norovirus GII.17, Guangdong Province, China, 2014–2015. Emerg Infect Dis 21:1240–1242. doi:10.3201/eid2107.150226. 26080037 PMC4480401

[5] Fu J, Ai J, Jin M, Jiang C, Zhang J, Shi C, Lin Q, Yuan Z, Qi X, Bao C, Tang F, Zhu Y 2015 Emergence of a new GII.17 norovirus variant in patients with acute gastroenteritis in Jiangsu, China, September 2014 to March 2015. Eurosurveillance 20:21157. doi:10.2807/1560-7917.ES2015.20.24.21157. 26111236

[6] Lee CC, Feng Y, Chen SY, Tsai CN, Lai MW, Chiu CH 25 August 2015 Emerging norovirus GII.17 in Taiwan. Clin Infect Dis [Epub ahead of print.] doi:10.1093/cid/civ647. 26306886

[7] Parra GI, Green KY 2015 Genome of emerging norovirus GII.17, United States, 2014. Emerg Infect Dis 21:1477–1479. doi:10.3201/eid2108.150652. 26196235 PMC4517714

[8] De Graaf M, van Beek J, Vennema H, Podkolzin A, Hewitt J, Bucardo F, Templeton K, Mans J, Nordgren J, Reuter G, Lynch M, Rasmussen L, Iritani N, Chan M, Martella V, Ambert-Balay K, Vinjé J, White P, Koopmans M 2015 Emergence of a novel GII.17 norovirus—end of the GII.4 era? Eurosurveillance 20:21178. doi:10.2807/1560-7917.ES2015.20.26.21178. 26159308 PMC5921880

[9] Eden J-S, Tanaka MM, Boni MF, Rawlinson WD, White PA 2013 Recombination within the pandemic norovirus GII.4 lineage. J Virol 87:6270–6282. doi:10.1128/JVI.03464-12. 23536665 PMC3648122

[10] Chan MCW, Leung TF, Chung TWS, Kwok AK, Nelson EAS, Lee N, Chan PKS 2015 Virus genotype distribution and virus burden in children and adults hospitalized for norovirus gastroenteritis, 2012–2014, Hong Kong. Sci Rep 5:11507. doi:10.1038/srep11507. 26082165 PMC4469980

[11] Chan MCW, Leung TF, Kwok AK, Lee N, Chan PK, Neuwirth R, Majeed M, Woodward WB, Musser JM 2014 Characteristics of patients infected with norovirus GII.4 Sydney 2012, Hong Kong, China. Emerg Infect Dis 20:658–661. doi:10.3201/eid2004.131457. 24656073 PMC3966374

